# Uterine extracellular matrix components are altered during defective decidualization in interleukin-11 receptor α deficient mice

**DOI:** 10.1186/1477-7827-2-76

**Published:** 2004-11-10

**Authors:** Christine A White, Lorraine Robb, Lois A Salamonsen

**Affiliations:** 1 Prince Henry's Institute of Medical Research, Clayton, Victoria 3168, Australia; 2 Dept of Obstetrics & Gynaecology, Monash University, Clayton, Victoria 3168, Australia; 3 Walter and Eliza Hall Institute of Medical Research, Melbourne, Victoria 3001, Australia

## Abstract

**Background:**

Implantation of the embryo and successful pregnancy are dependent on the differentiation of endometrial stromal cells into decidual cells. Female interleukin-11 receptor α (IL-11Rα) deficient mice are infertile due to disrupted decidualization, suggesting a critical role for IL-11 and its target genes in implantation. The molecular targets of IL-11 in the uterus are unknown, but it is likely that IL-11 signaling modifies the expression of other genes important in decidualization. This study aimed to identify genes regulated by IL-11 during decidualization in mouse uterus, and to examine their expression and localization as an indication of functional significance during early pregnancy.

**Methods:**

Decidualization was artificially induced in pseudopregnant wild type (*IL11Ra*^+/+^) and IL-11Rα deficient (*IL11Ra*^-/-^) littermates by oil injection into the uterine lumen, and gene expression analyzed by NIA 15K cDNA microarray analysis at subsequent time points. Quantitative real-time RT-PCR was used as an alternative mRNA quantitation method and the expression and cellular localization of the protein products was examined by immunohistochemistry.

**Results:**

Among 15,247 DNA probes, 13 showed increased and 4 decreased expression in *IL11Ra*^-/-^ uterus at 48 h of decidualization. These included 4 genes encoding extracellular matrix proteins; collagen III α1, secreted acidic cysteine-rich glycoprotein (SPARC), biglycan and nidogen-1 (entactin). Immunohistochemistry confirmed increased collagen III and biglycan protein expression in *IL11Ra*^-/-^ uterus at this time. In both *IL11Ra*^-/-^ and wild type uterus, collagen III and biglycan were primarily localized to the outer connective tissue and smooth muscle cells of the myometrium, with diffuse staining in the cytoplasm of decidualized stromal cells.

**Conclusion:**

These data suggest that IL-11 regulates changes in the uterine extracellular matrix that are necessary for decidualization.

## Background

Implantation of the embryo, formation of the placenta and successful pregnancy are dependent on the proliferation and differentiation of endometrial stromal cells into decidual cells. Decidualization occurs in response to endometrial and embryonic signals and is thought to involve complex interactions between ovarian steroid hormones, the uterine extracellular matrix (ECM), growth factors and cytokines (reviewed in [1]). When implantation is initiated at day 3.5 of pregnancy in the mouse (plug is day 0), decidualization begins adjacent to the embryo on the antimesometrial side of the uterus to form the primary decidual zone [2]. Decidual transformation then extends mesometrially to form the secondary decidual zone by day 6, accompanied by a dramatic increase in vascular permeability [3]. The endometrial response to implantation can be induced artificially by the application of oil into the lumen of the hormonally primed uterus, producing a deciduoma [4]. Natural and artificial decidualization share many of the same features, with distinct decidual zones [5], and the progression of each response can be monitored by an increase in uterine weight [6].

Female mice with a null mutation in the gene encoding interleukin-11 receptor α (IL-11Rα) are infertile due to disrupted decidualization [7], suggesting a critical role for IL-11 and its target genes in the decidual response. Despite normal estrous cycles and no detectable ovarian defects, female IL-11Rα deficient (*IL11Ra*^-/-^) mice are unable to support implantation of either *IL11Ra*^-/- ^or wild type embryos. The failure of decidualization between days 4.5 and 10.5 in *IL11Ra*^-/- ^females is characterized by greatly reduced vascular permeability at implantation sites, areas of hemorrhage, impaired secondary decidual zone formation, absence of mesometrial decidualization and aberrant infiltration of trophoblast giant cells [7]. Although morphologically similar to the decidua of pregnancy, a minority of artificially induced deciduomata in *IL11Ra*^-/- ^mice show some mesometrial decidualization. Females homozygous for a hypomorphic IL-11Rα allele also show reduced decidualization, with decreased cell proliferation, progressive degeneration of the deciduae, infiltration of trophoblast giant cells and absence of placental formation [8]. Neither of these mutations have been found to cause hematopoietic defects [8,9].

Interleukin-11 is a multifunctional cytokine, initially described as a bone marrow stroma-derived hematopoietic growth factor [10]. IL-11 shares many functions with other members of the IL-6 family of cytokines, including the induction of acute phase proteins [11], inhibition of adipogenesis [12] and the regulation of bone ECM metabolism via induction of tissue inhibitor of metalloproteinases (TIMP)-1 [13]. Like IL-6, leukemia inhibitory factor (LIF), oncostatin M, ciliary neurotrophic factor and cardiotrophin-1, IL-11 exerts its biological effects via a multisubunit receptor complex involving the signal transducer gp130 [14]. Following the formation of its hexameric receptor composed of two molecules each of IL-11, the low-affinity ligand-binding IL-11Rα and gp130 [15], IL-11 is capable of activating a number of downstream signaling pathways. In most cell types, IL-11-activated gp130 mediates its effects through Janus tyrosine kinases (JAKs1-3 and Tyk2) and the signal transducers and activators of transcription (STATs1-6) (reviewed in [16]). The rate of transcription of target genes is then modified by binding of activated STAT dimers to a DNA element in the promoter region. IL-11 signaling can also control the initiation of translation via sequential activation of PI3-K, Pdk-1/Akt, p70 S6 kinase and ribosomal protein S6 [17].

Localization and expression of IL-11, IL-11Rα and gp130 in human endometrium across the menstrual cycle suggests a role for this cytokine in decidual transformation in preparation for pregnancy [18-20]. Levels of immunoreactive IL-11 are highest during the secretory phase of the cycle, when the endometrium is receptive to implantation, and IL-11 is produced by the decidualized stromal cells. Treatment of human endometrial stromal cells in culture with recombinant human IL-11 increases their secretion of the decidual markers prolactin and insulin-like growth factor binding protein (IGFBP)-1, and is associated with enhanced differentiation [21]. Plasma levels of IL-11 are decreased in women with first trimester spontaneous abortion [22], and there is decreased expression of IL-11 protein in chorionic villi and decidua from anembryonic compared to normal pregnancy [23].

The molecular targets of IL-11 in the uterus are unknown, but it is likely that IL-11 signaling modifies the expression of other genes important in decidualization. This study aimed to identify genes regulated by IL-11 during decidualization by cDNA microarray, and to examine their expression and localization by immunohistochemistry, as an indication of functional significance during early pregnancy.

## Methods

### Animals

Mice deficient in IL-11 receptor α (*IL11Ra*^-/-^) had been previously generated by gene targeting, and serially crossed more than 10 generations onto a C57BL/6 background [9]. Heterozygotes were interbred to produce wild-type (*IL11Ra*^+/+^) and IL-11Rα deficient (*IL11Ra*^-/-^) mice, which were identified by Southern blot analysis of genomic DNA obtained from tail biopsies [9]. All mice were housed in conventional conditions, fed and watered ad libitum and maintained in a 12-h light, 12-h dark cycle. All procedures were approved by the Monash Medical Centre (B) Animal Ethics Committee (AEC# MMCB 2001/04), and were carried out in compliance with the Helsinki Declaration.

### Artificial decidualization

Surgery was performed under xylazine/ketamine-induced anesthesia, by intraperitoneal administration of 10 mg/kg xylazine hydrochloride (Ilium Xylazil-20, Troy Laboratories, Smithfield, NSW, Australia) and 80 mg/kg ketamine hydrochloride (Ketalar, Pfizer, West Ryde, NSW, Australia) in sterile phosphate-buffered saline (PBS). Anesthesia was reversed with 250 μg yohimbine hydrochloride and 400 μg 4-amino pyridine (Reverzine S.A., Parnell Laboratories, Alexandria, NSW, Australia) in sterile PBS.

Female *IL11Ra*^+/+ ^and *IL11Ra*^-/- ^littermates at 8–12 weeks of age (n = 4 per genotype per time point) were mated with wild type vasectomized males to induce pseudopregnancy, with the day of plug detection designated day 0. At approximately 1400 h on day 3, decidualization was induced throughout both uterine horns of each animal by injection of 20 μl of sesame oil (Sigma Chemical Co., St Louis, MO) into the lumen of each uterine horn via a 26-gauge needle inserted just distal to the utero-tubal junction. Mice were necropsied prior to surgery (0 h) or at 18 h, 24 h or 48 h following artificial decidualization. These time points prior to the onset of secondary decidualization were chosen to ensure similar cellular composition of the *IL11Ra*^+/+ ^and *IL11Ra*^-/- ^uteri. Whole uteri were cleaned of fat and weighed. A section of each uterus was either fixed in 10% phosphate-buffered formalin overnight or Carnoy's fixative for 2 h and processed to paraffin wax, or snap frozen in liquid nitrogen for subsequent RNA isolation.

### Statistical analysis of uterine weight data

The wet weights of whole uterus from *IL11Ra*^+/+ ^(n = 4/time point) and *IL11Ra*^-/- ^(n = 4/time point) mice were statistically analyzed using GB-Stat 6.5 (Dynamic Microsystems, Inc., Silver Spring, MD). Following Bartlett's test for homogeneity of variance, uterine weight was used as the dependent variable and genotype and time as the two independent variables in a two factor analysis of variance. Bonferroni multiple comparison testing was used to compare uterine weight across time in each genotype and between genotypes at each time point. A two-tailed *p *value of less than 0.05 was considered a significant difference.

### RNA preparation, cDNA microarray hybridization and data collection

Total RNA was extracted from whole uterus by acid guanidinium thiocyanate-phenol-chloroform extraction [24], incorporating an additional chloroform purification step to remove contaminating phenol. RNA was then treated with ribonuclease (RNase)-free deoxyribonuclease (DNase; Ambion, Austin, TX) to remove genomic DNA. The concentration of RNA in the final preparation was determined spectrophotometrically, and RNA quality evaluated by gel electrophoresis (1.2% agarose; Roche Applied Science, Penzberg, Germany) and by the ratio of optical density (OD_260_:OD_280 _= 1.8–2.0). Each artificially decidualized *IL11Ra*^+/+ ^or *IL11Ra*^-/- ^uterus was processed individually for microarray hybridization. A reference pool of RNA was prepared from wild type unstimulated uteri (n = 16). The experimental design used indirect comparisons between *IL-11Ra*^+/+ ^or *IL11Ra*^-/- ^and the reference pool.

Total RNA (10 μg) served as a template for the synthesis of aminoallyl-cDNA, which was then coupled to a fluorescent dye ester (Cy3 or Cy5) as described by the manufacturers of the CyScribe cDNA Post Labelling Kit (Amersham Biosciences, Buckinghamshire, England). Microcon-30 size exclusion columns (Millipore, Billerica, MA) were used to initially concentrate RNA samples, and to purify the cDNA probes prior to and following the dye-coupling reaction. Slides were printed with sequence-verified duplicate spots of the NIA 15K cDNA clone set [25] at the Australian Genome Research Facility and prehybridized for 30 min at 42 C in 10 mg/ml bovine serum albumin (BSA, ICN Biomedicals, Aurora, OH), 25% formamide (BDH Laboratory Supplies, Poole, England), 5 × SSC (750 mM sodium chloride, 75 mM sodium citrate; BDH Chemicals/AnalaR, Kilsyth, Victoria, Australia) and 0.1% sodium dodecyl sulfate (SDS; BDH Chemicals/AnalaR). *IL11Ra*^+/+ ^or *IL11Ra*^-/- ^cDNA (Cy3) and reference cDNA (Cy5) were competitively hybridized to the microarrays in the presence of 1 mg/ml Cot1 DNA (Gibco-BRL, Life Technologies, Mount Waverley, Australia), 10 mg/ml salmon sperm DNA (Gibco-BRL) and 10 mg/ml polyadenylic acid (Sigma). Hybridizations were repeated using the alternate dye combinations to account for differential fluorescent dye incorporation. After washing, slides were scanned using an Axon GenePix 4000B microarray reader (Axon Instruments, Union City, CA) and GenePix Pro 4.0 software (Axon Instruments) to generate pairs of 16-bit tagged image file format (TIFF) files. Following manual quality control for hybridization artefacts, red (Cy5) and green (Cy3) mean foreground and median background fluorescence intensity measurements for each spotted DNA sequence were extracted for export to the statistical programming environment R 1.5.1.

### Microarray data analysis

Differential gene expression between *IL11Ra*^+/+ ^(WT) and *IL11Ra*^-/- ^(KO) uterus at time points following artificial decidualization was determined using the normalization and analysis functions of the statistical language R [26] and the add-on package Statistics for Microarray Analysis [27]. For each time point, the red and green background-corrected intensities (*R *and *G*) from 4 slides were read into R using the read.genepix and init.data commands. The stat.ma function was used to calculate the log-ratios of expression (*M *= log_2_*R *- log_2_*G*) and average log-intensity (A = (log_2_*R *+ log_2_*G*)/2) for each spot, and to normalize the red and green channels relative to one another using print-tip loess normalization [28]. Diagnostic *MA *plots of each slide were used to determine the effectiveness of this normalization method in adjusting for sources of variation arising from dye bias and print-tip effects.

Given the indirect dye swap design [29] of these experiments, log-ratio values were reversed in the dye-swapped slides and the log-ratio for each gene was calculated as the difference of two independent log ratios from the equation log (KO/WT) = log (KO/reference) - log (WT/reference). Differentially expressed genes were identified by considering a univariate testing problem for each gene and then correcting for multiple testing using adjusted p-values [30]. The function stat.lm [31] was used to fit a linear model for each gene to the series of 4 arrays and estimate the average fold change and a standard deviation for each gene, taking into account the pattern of dye swaps and duplicate spots. The stat.bay.est command [31] then computed a moderated *t*-statistic and *B*-statistic for each gene, to give a log odds ratio of difference in mRNA expression between *IL11Ra*^+/+ ^and *IL11Ra*^-/-^. Genes with a log odds score of greater than 3 (ie. adjusted p-value < 0.05) in both replicates were considered to be significantly up- or down-regulated in *IL11Ra*^-/- ^uterus compared to wild type.

### Real-time RT-PCR

Total RNA samples extracted from *IL11Ra*^+/+ ^(n = 2) and *IL11Ra*^-/- ^(n = 2) uterus at 48 h of decidualization and used for microarray analysis, and an additional two samples per genotype prepared in the same way were used for validation of the microarray data by real-time reverse transcription polymerase chain reaction (RT-PCR). All samples (n = 4/genotype) were further purified through an RNeasy Spin Column (Qiagen, Valencia, CA), and quantitated by RiboGreen Assay (Molecular Probes, Eugene, OR) according to the manufacturer's instructions, prior to triplicate reverse transcription reactions.

Total RNA (1 μg) was reverse transcribed at 46 C for 1.5 h in 20 μl reaction mixture using 100 ng random hexanucleotide primers and 6 IU AMV reverse transcriptase (Roche, Castle Hill, Australia) in the presence of cDNA synthesis buffer (Roche), 1 mM dNTPs (Roche), 10 mM dithiothreitol (Roche) and 10 IU ribonuclease inhibitor (RNasin; Promega, Annandale, Australia). The resulting cDNA mixtures were heated at 95 C for 5 min before storage at -20 C in small volumes to avoid freeze-thawing. Negative controls were performed by omission of reverse transcriptase.

For real-time quantification of selected mRNA transcript levels in *IL11Ra*^+/+ ^and *IL11Ra*^-/- ^uterus at 48 h of decidualization, PCR was carried out using a Roche LightCycler. Prior to LightCycler analysis, standard cDNA for each gene of interest was generated using a PCR Express block cycler (Thermo Hybaid Instruments, Franklin, MA). A 1 μl aliquot of RT product was amplified in a total volume of 40 μl using 4 μl of 10 × PCR Reaction Buffer (100 mM Tris-HCl, 15 mM MgCl_2_, 500 mM KCl, pH 8.3; Roche), 62.5 μM dNTPs (Gibco-BRL, Life Technologies), 10 pmol sense and antisense primers (Sigma Genosys, Castle Hill, NSW, Australia; Table 1) and 2.5 IU Taq DNA polymerase (Roche). The PCR amplification consisted of a hot start at 95 C for 5 min followed by 35 – 40 cycles of denaturation at 94 C for 50 sec, annealing at x C (see annealing temperature in Table 2) for 40 sec and extension at 72 C for 40 sec. The final extension was performed at 72 C for 10 min. Optimal annealing temperature (x) and cycle number were determined for each primer pair (Table 2). PCR products were electrophoresed on a 1.5% agarose gel containing 200 ng/ml ethidium bromide. Each single amplified product band was excised from the gel and purified using the UltraClean GelSpin DNA Extraction Kit (Mo Bio Laboratories, Solana Beach, CA). The cDNA concentration was measured using a spectrophotometer and each sequence identity confirmed at the Wellcome Trust Sequencing Centre, Monash Medical Centre. Standard curves for each transcript were generated using serial 1:10 dilutions of this standard cDNA using sterile water. Samples were diluted 1:10 – 1:40 prior to LightCycler analysis. Absolute concentrations of mRNA present in *IL11Ra*^+/+ ^and *IL11Ra*^-/- ^uterus at 48 h of decidualization were calculated relative to the standard curve, and adjusted for 18S rRNA expression levels.

**Table 1:** Oligonucleotide primers used in real-time RT-PCR. Primer pairs previously published – COL3A1 [82], BGN [83], SPARC [84] and NID1 [85].

**Target**	**Sense primer sequence**	**Antisense primer sequence**
COL3A1	5'-GGCTCCTGGTGAGCGAGGACG-3'	5'-CCCATTTGCACCAGGTTCTCC-3'
BGN	5'-CGGGACCTTGCTGTCTTCTC-3'	5'-CCCGGCAAGAACCTGAAAG-3'
SPARC	5'-AGAAGGCCTTTAGCCCTCTGC-3'	5'-ACTTTGCGGATACGGTTGTC-3'
NID1	5'-AGCTTCTATGATCGTACGGACATCAC-3'	5'-GTAAAGAACTGTAGACCATCTTCAGG-3'
18S	5'-CGGCTACCACATCCAAGGAA-3'	5'-GCTGGAATTACCGCGGCT-3'

**Table 2:** Real-time RT-PCR amplification conditions for each primer pair

**Primer pair**	**PCR product size (bp)**	**Annealing temp (C)**	**No. of cycles**	**[MgCl_2_] (mmol/l)**	**Melting temp of PCR product (C)**
COL3A1	522	64	40	3	91.2
BGN	131	58	40	5	86.3
SPARC	130	60	40	3	85.6
NID1	425	63	40	4	88.8
18S	187	60	35	4	86.8

The cDNA template (triplicate RT reactions for each of 4 animals per genotype; 4 μl) was added to sterile capillaries to a total volume of 20 μl containing SYBR Green I, dNTPs, Taq DNA polymerase and reaction buffer (LightCycler FastStart DNA Master SYBR Green I Kit; Roche), supplemented with 5 pmol of specific sense and antisense primers (Table 1) and an optimal concentration of MgCl_2 _(Table 2). An initial denaturing step was performed for 10 min at 95 C, prior to 35 – 40 cycles of 95 C for 15 sec, x C for 5 sec and 72 C for 10 sec. Fluorescence was monitored continuously during cycling at the end of each elongation phase. At the end of each program, melting curve analysis confirmed the specificity of the reaction products.

### Statistical analysis of real-time RT-PCR data

Triplicate RT reactions for each sample, the standard curve and a no RT negative control were analyzed in the same run, and each run was repeated once. A mean value for the initial target concentration of each sample was calculated using the fit points function of the LightCycler software. The mean concentration of 18S rRNA for each sample was used to control for RNA input, as it is considered a stable housekeeping gene and was not altered in expression between *IL11Ra*^+/+ ^and *IL11Ra*^-/- ^uterus. Following normalization, levels of RNA for COL3A1, BGN, SPARC and NID1 in *IL11Ra*^-/- ^compared to wild type were statistically analysed using the paired t-test function of GraphPad Prism 3.0 (GraphPad Software, San Diego, CA). A two-tailed *p *value of less than 0.05 was considered a significant difference.

### Immunohistochemistry

To confirm differential expression of collagen III, biglycan, SPARC and nidogen-1 at the protein level, immunohistochemistry was carried out on transverse sections of *IL11Ra*^+/+ ^and *IL11Ra*^-/- ^uterus collected at 48 h of decidualization, using specific antibodies (n = 5 mice per genotype, n = 3 sections per mouse). The morphology and cellular localization of artificial decidualization in *IL11Ra*^+/+ ^and *IL11Ra*^-/- ^uterus was also determined by immunostaining for the decidual marker desmin [32]. Primary antibodies used were rabbit anti-mouse collagen type III (Abcam #ab7778, Cambridge, UK) at 5 μg/ml, rabbit anti-mouse biglycan (LF-159 [33], gift from Dr. Larry Fisher, Matrix Biochemistry Unit, National Institutes of Health, Bethesda, MD) at 1:1000 dilution of whole serum, goat anti-mouse SPARC (Santa Cruz Biotechnology #sc13326, Santa Cruz, CA) at 5 μg/ml, rat anti-mouse entactin/nidogen-1 (Lab Vision-NeoMarkers #RT797P, Fremont, CA) at 15 μg/ml and goat anti-mouse desmin (Santa Cruz) at 200 μg/ml. Secondary antibodies were biotinylated swine anti-rabbit immunoglobulin G (IgG, DAKO, Glostrup, Denmark; 1:200), horse anti-goat IgG (Vector Laboratories, Burlingame, CA; 1:100) and rabbit anti-rat IgG (DAKO; 1:200). For collagen III, SPARC, nidogen-1 and desmin, negatives were performed using a matching concentration of non-immune IgG of the species in which the primary antibody was raised; rabbit IgG (DAKO), goat IgG (R&D Systems, Minneapolis, MN) or rat IgG (DAKO). For biglycan, negatives were performed using a matching dilution of normal rabbit serum (Sigma). Mouse lung (collagen III and biglycan) and kidney (nidogen-1 and SPARC) were used as positive control tissues, and a section from a single block was included in each staining run for quality control. For collagen III and SPARC immunolocalization, all dilutions and washes were carried out in high salt Tris-buffered saline (HS-TBS, 300 mM NaCl, 5 mM TrisHCl) with 0.6 % Tween 20. TBS (150 mM NaCl, 5 mM TrisHCl) with 0.6% Tween was used for nidogen-1, TBS/0.3% Tween for biglycan and HS-TBS/0.1% Tween for desmin.

Five micron sections of Carnoy's-fixed (for collagen III and biglycan immunolocalization) or formalin-fixed (for SPARC, nidogen-1 and desmin) uterus were mounted on poly-L-lysine-coated glass slides, deparaffinized and rehydrated through a series of graded ethanols. In an humidified chamber at 25 C, sections were incubated for 10 min in 3% hydrogen peroxide to block endogenous peroxidase activity, then for 30 min (SPARC and desmin) or 1 h (collagen III, biglycan and nidogen-1) in 10% serum of the species in which the secondary antibody was raised (swine, horse or rabbit; Sigma) and 2% mouse serum in one of the above buffers. Sections were then incubated with primary antibody or negative at 4 C overnight (collagen III, biglycan, nidogen-1 and SPARC) or 30 min at 25 C (desmin), then washed in TBS/Tween prior to secondary antibody incubation for 30 min (SPARC and desmin) or 1 h (collagen III, biglycan and nidogen-1) at 25 C. Sections were again washed in TBS/Tween, then the secondary antibody detected using the Vectastain ABC Elite/HRP Kit (collagen III and nidogen-1; Vector Laboratories) or the StreptABComplex/HRP Kit (biglycan, SPARC and desmin; DAKO) according to the manufacturer's instructions. Protein localization was visualized using the Liquid DAB-Plus Substrate Chromogen System (DAKO), with Harris hematoxylin (Sigma) counterstain.

## Results

### Artificial decidualization of *IL-11Rα *deficient and wild type uterus

**Figure 1:**
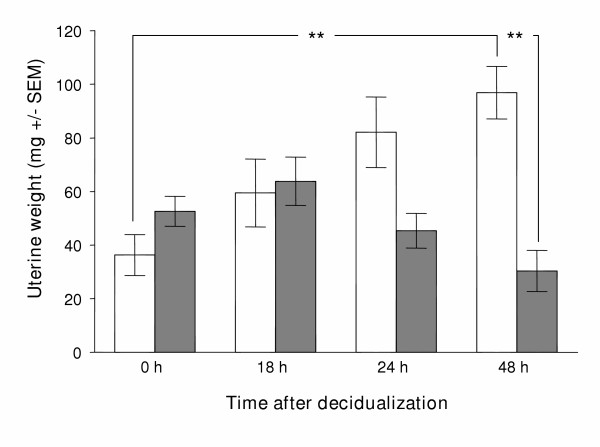
**Uterine weight following artificial decidualization. **Weight (mg +/- SEM) of uterine horns at times following artificial decidualization of *IL11Ra*^+/+ ^(open bars) and *IL11Ra*^-/- ^(shaded bars) littermates. ** *p *< 0.01.

Following the injection of oil into the wild type pseudopregnant uterus, a progressive increase in uterine weight was observed from 0 through to 48 h, reaching statistical significance (*p *< 0.01) at the final time point (Fig. 1). In contrast, the weight of the artificially decidualized *IL11Ra*^-/- ^uterus did not change significantly across consecutive time points. There was therefore a statistically significant difference (*p *< 0.01) in uterine weight at 48 h of artificial decidualization between *IL11Ra*^+/+ ^(96.9 +/- 9.8 mg) and *IL11Ra*^-/- ^(30.4 +/- 7.7 mg).

### Differential gene expression following artificial decidualization

**Figure 2:**
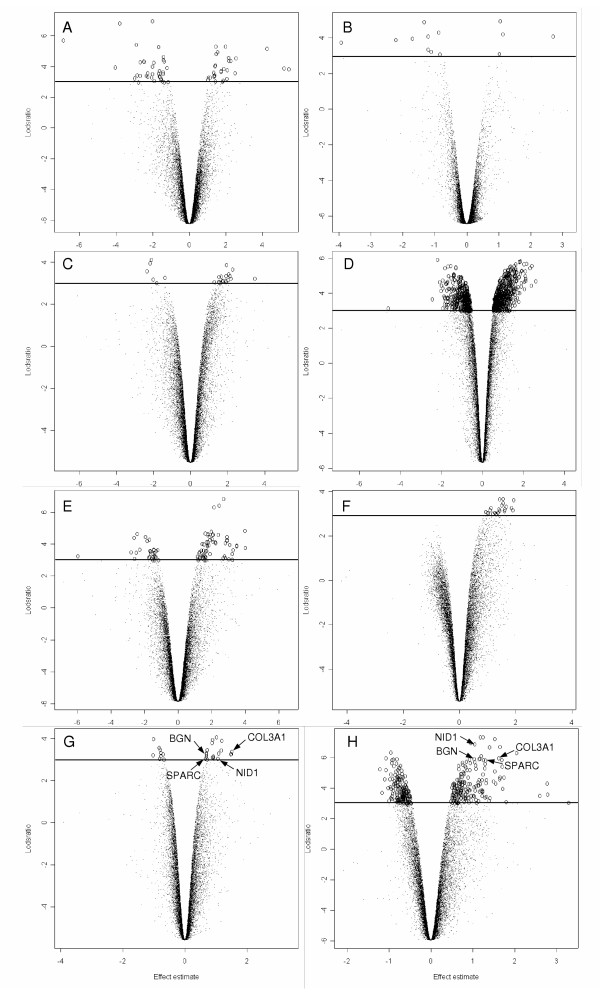
**Gene expression in *IL11Ra*^+/+ ^and *IL11Ra*^-/- ^uterus following artificial decidualization. **Expression profiling of 15K genes between *IL11Ra*^+/+ ^and *IL11Ra*^-/- ^at 0 h (A, B), 18 h (C, D), 24 h (E, F) and 48 h (G, H) following the artificial induction of decidualization. Each volcano style plot shows the normalized log ratio (effect estimate) of *IL11Ra*^-/- ^compared to wild type for each gene from a series of 4 microarrays, plotted against the log odds of differential expression. A, C, E, G represent the first replicates, and B, D, F, H the second dye-swapped replicates. Genes with log odds of differential expression greater than 3 (ie. adjusted *p*-value < 0.05, above horizontal line) are represented by open circles, and COL3A1, BGN, SPARC and NID1 are labeled in G and H. Only those genes with log odds of differential expression greater than 3 in both replicates were considered differentially expressed, as described in *Methods*.

Total RNA extracted from *IL11Ra*^+/+ ^and *IL11Ra*^-/- ^uterus artificially decidualized for 0, 18, 24 or 48 h (n = 2/genotype/time point) was used as a template for the hybridization of NIA 15K cDNA microarrays. Figure 2 shows the volcano style plots of the normalized data for all genes at each time point. Each plot summarizes the data for a series of 4 microarrays (two KO/REF and two WT/REF), with differentially expressed genes in each replicate represented by open circles above the horizontal line (*p *= 0.05). At 0 h, prior to application of the decidualizing stimulus on day 3 of pseudopregnancy, there were no reproducible differentially expressed genes between *IL11Ra*^+/+ ^and *IL11Ra*^-/- ^(Fig. 2A,2B). Following 18 h of decidualization (Fig 2C,2D; Table 3), five expressed sequence tags (ESTs) were consistently upregulated 2 – 3-fold in *IL11Ra*^-/- ^uterus compared to wild type. At 24 h of decidualization (Fig. 2E,2F; Table 4), there was one EST upregulated 2.7-fold. Sequence information for these ESTs is available online [34], using the AGRF ID as a unique identifier. None of these ESTs are currently recognized as sharing strong homology to any known genes. At 48 h of decidualization, 13 cDNAs showed upregulation and 4 downregulation in IL-11Rα deficient uterus (Fig. 2G,2H; Table 5). A number of these genes have previously described roles in the endometrium, but prior to this study, none have been shown to interact with IL-11. The ECM genes COL3A1, SPARC, BGN and NID1 were among those upregulated in *IL11Ra*^-/- ^uterus compared to wild type. Transcripts representing COL3A1 and SPARC were present at two different locations on the array, and in each case, both sets of duplicate spots showed consistent upregulation in the absence of IL-11Rα. There were no genes or ESTs which were differentially expressed at more than one time point.

**Table 3:** Differentially expressed genes in *IL11Ra*^-/- ^uterus compared to wild type at 18 h of decidualization

**AGRF ID**	**GenBank Accession**	**UniGene Cluster**	**Gene**	**Fold Change**	**P Value**
H3084H06	BG070211	Mm.25335	EST: Cdc14b: CDC14 cell division cycle 14 homolog B (S. cerevisiae)	+ 3.1	0.044
H3137G08	BG074662	Mm.197274	EST: Moderately similar to GNMSLL retrovirus-related reverse transcriptase homolog – mouse retrotransposon (M. musculus)	+ 3.0	0.046
H3123E03	BG073512	Mm.197252	EST: Weakly similar to TLM MOUSE TLM PROTEIN (M. musculus)	+ 2.9	0.047
H3073G12	BG069200	Mm.328026	EST: Transcribed sequence with moderate similarity to protein ref:NP_083358.1 (M. musculus); RIKEN cDNA 5830411J07	+ 2.5	0.038
H3082G05	AU014577	Not assigned	EST	+ 2.1	0.032

**Table 4:** Differentially expressed genes in *IL11Ra*^-/- ^uterus compared to wild type at 24 h of decidualization

**AGRF ID**	**GenBank Accession**	**Unigene Cluster**	**Gene**	**Fold Change**	**P Value**
H3091F10	BG064439	Mm.182580	EST: Transcribed sequence with weak similarity to protein ref:NP_081764.1 (M. musculus); RIKEN cDNA 5730493B19	+ 2.7	0.027

**Table 5:** Differentially expressed genes in *IL11Ra*^-/- ^uterus compared to wild type at 48 h of decidualization

**AGRF ID**	**GenBank Accession**	**Unigene Cluster**	**Gene**	**Fold Change**	**P Value**
H3012B10	BG063737	Mm.28870	45S pre rRNA gene	+ 4.2	0.002
H3133G03	BG074327	Mm.147387	Procollagen III alpha-1 (COL3A1)	+ 3.0	0.010
H3124H10	BG073709	Mm.147387	Procollagen III alpha-1 (COL3A1)	+ 2.7	0.011
H3116A04	BG072874	Mm.35439	Secreted acidic cysteine rich glycoprotein (SPARC/osteonectin/BM-40)	+ 2.4	0.010
H3152F04	BG075853	Mm.22699	Selenoprotein P plasma 1 (SEPP1)	+ 2.3	0.004
H3023A11	BG064718	Mm.21228	EST: RIKEN cDNA 2610101J03 gene; expressed sequence C79684	+ 2.2	0.038
H3024A05	BG064802	Mm.35439	Secreted acidic cysteine rich glycoprotein (SPARC/osteonectin/BM-40)	+ 2.2	0.027
H3128D02	BG073888	Mm.77432	Thioredoxin interacting factor (VDUP1)	+ 2.2	0.003
Hs.ALAD	BC000977	Hs.1227	Aminolevulinate delta dehydratase (ALAD)	+ 2.0	0.028
H3127D03	BG073809	Mm.2608	Biglycan (BGN/PGI)	+ 1.8	0.010
H3025E04	BG064933	Mm.4691	Nidogen-1 (NID1/entactin)	+ 1.8	0.007
H3129D02	BG073972	Mm.2137	Transcriptional regulator SIN3 yeast homolog B	+ 1.7	0.014
H3078E09	BG069642	Mm.27816	Hexosaminidase B (HEXB)	+ 1.7	0.013
H3101C10	BG071626	Mm.161419	Glyceraldehyde-3-phosphate dehydrogenase (GAPDH)	- 1.6	0.015
H3122C04	BG073406	Mm.68999	EST: RIKEN cDNA 9430015G10 gene	- 1.6	0.036
H3116E07	BG072919	Mm.172198	EST: Weakly similar to GNMSLL retrovirus-related reverse transcriptase homolog – mouse retrotransposon (M. musculus)	- 2.0	0.032
H3144C05	BG075183	Mm.337732	EST	- 2.1	0.014

### Validation of gene expression by real-time RT-PCR

**Figure 3:**
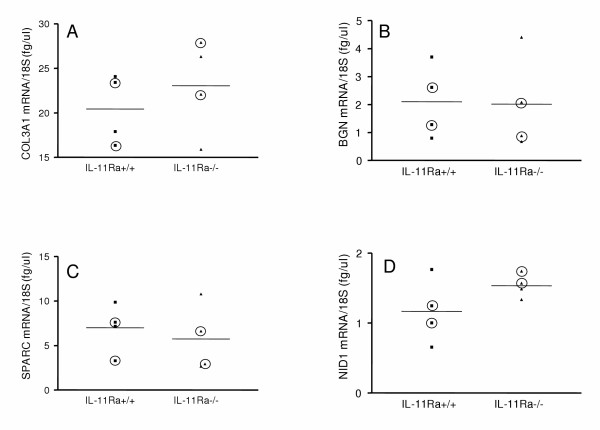
**Quantitative real-time RT-PCR for extracellular matrix components. **Quantitative real-time RT-PCR for (A) COL3A1, (B) BGN, (C) SPARC and (D) NID1. Circled data points indicate samples used in the cDNA microarray analysis, and horizontal lines the mean of each genotype. Absolute values for mRNA abundance were normalized to that of 18S rRNA.

To confirm the altered mRNA expression of the ECM genes COL3A1, SPARC, BGN and NID1 at 48 h of decidualization, quantitative real-time RT-PCR was carried out using the same RNA samples used in the microarray analysis, plus two additional RNA samples of each genotype, collected in the same way. At a significance level of *p* < 0.05, there was no statistical difference in the abundance of 18S rRNA (data not shown), COL3A1 (Fig. 3A), BGN (Fig. 3B), SPARC (Fig. 3C) or NID1 (Fig. 3D) mRNA between *IL11Ra*^+/+ ^and *IL11Ra*^-/- ^uterus. When only the samples used in the microarray analysis were considered, the difference in NID1 abundance between *IL11Ra*^+/+ ^(1.13 +/- 0.12 fg/μl) and *IL11Ra*^-/- ^(1.66 +/- 0.09 fg/μl) uterus approached statistical significance at *p* = 0.0708.

### Validation of gene expression by immunohistochemistry

Four genes found to be differentially expressed in *IL11Ra*^-/- ^uterus compared to wild type at 48 h of decidualization were investigated at the protein level by immunohistochemistry using specific antibodies. Decidualizing and fully decidualized cells were identified in adjacent sections by immunostaining for the intermediate filament protein desmin, well characterized as a marker for decidual transformation [32].

**Figure 4:**
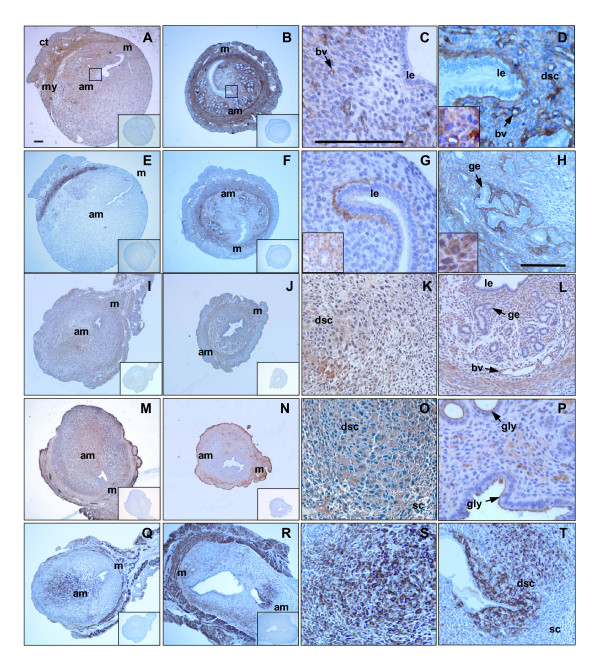
**Immunohistochemistry for extracellular matrix components. **Immunohistochemical staining of wild type (A, C, E, G, I, K, M, O, P, Q, S) and *IL11Ra*^-/- ^(B, D, F, H, J, L, N, R, T) uterus at 48 h of decidualization using specific antibodies for collagen III (A, B, C, D), biglycan (E, F, G, H), nidogen-1 (I, J, K, L), SPARC (M, N, O, P) and desmin (Q, R, S, T). Negative controls using a matching concentration of non-immune IgG (collagen III, nidogen-1, SPARC and desmin) or normal serum (biglycan) in place of the primary antibody are inset in A, B, E, F, I, J, M, N, Q and R. Black squares on A and B indicate the antimesometrial pole magnified in C and D. Abbreviations: connective tissue (ct), myometrium (my), mesometrial pole (m), antimesometrial pole (am), luminal epithelium (le), glandular epithelium (ge), decidualized stromal cell (dsc), non-decidualized stromal cell (sc), blood vessel (bv), glycocalyx (gly). Scale bar = 50 μm (A, B, E, F, I, J, M, N, Q and R are at the same magnification; C, D, G and P are at the same magnification; H, K, L, O, S, T and inset in G are at the same magnification).

Microarray data showing highly significant and reproducible increases in COL3A1 and BGN mRNA levels in *IL11Ra*^-/- ^uterus were reflected in increased staining intensity for collagen III (Fig. 4A,4B,4C,4D) and biglycan (Fig. 4E,4F,4G,4H) in *IL11Ra*^-/- ^uterus (Fig. 4B,4D,4F,4H) compared to wild type (Fig. 4A,4C,4E,4G). In both *IL11Ra*^-/- ^and wild type uterus, collagen III and biglycan were primarily localized to the outer connective tissue and smooth muscle cells of the myometrium, with diffuse staining in the cytoplasm of decidualized stromal cells (Fig. 4D,4H inserts). Interstitial compartments underlying luminal and glandular epithelium and surrounding blood vessels also showed strong immunoreactivity for both proteins, while the epithelial cells were negative. In the absence of IL-11Rα, stronger staining for collagen III was particularly evident underlying luminal epithelium and in the ECM surrounding decidualizing stromal cells. There was a consistent absence of subluminal collagen III staining on the antimesometrial side of the uterus in wild type animals, an effect not seen in *IL11Ra*^-/- ^littermates (Fig. 4C,4D). There was also a clear difference in the localization of biglycan staining underlying luminal epithelium, with strong staining at the mesometrial pole of the uterus in wild type animals and no preferential localization to either pole in *IL11Ra*^-/- ^animals (Fig 4E,4F,4G). Biglycan staining surrounding glands was much more intense in *IL11Ra*^-/- ^uterus (Fig. 4H) compared to wild type (Fig. 4G insert).

While no detectable differences were observed in the overall intensity of immunostaining for nidogen-1 (Fig. 4I,4J,4K,4L) or SPARC (Fig. 4M,4N,4O,4P) in *IL11Ra*^-/- ^uterus compared to wild type, the localization of these proteins has not previously been described in the deciduoma of wild type or *IL11Ra*^-/- ^mice. In both genotypes, nidogen-1 was localized to the cytoplasm of decidual cells (Fig. 4K) and glandular epithelial cells (Fig. 4L), the basement membrane underlying luminal and glandular epithelium and surrounding blood vessels (Fig. 4L). The cellular localization of SPARC was more similar to that of collagen III and biglycan, with strong staining in the outer connective tissue and myometrium (Fig. 4M,4N). Strong SPARC staining was also detected in the cytoplasm of decidualized and non-decidualized stromal cells (Fig. 4O), endothelial cells (Fig. 4P), and at the glycocalyx of luminal and glandular epithelium (Fig. 4P).

Desmin immunostaining revealed a reduction in the overall extent of decidualization in *IL11Ra*^-/- ^uteri at 48 h following the induction of deciduomata (*IL11Ra*^+/+^: Fig. 4Q,4S; *IL11Ra*^-/-^: Fig. 4R,4T), with an absence of secondary decidualization. Desmin-positive decidual cells were detected in all artificially decidualized uteri, indicating that the surgical induction of decidualization was successful in all cases.

## Discussion

Interleukin-11 is one of only a few molecules known to be critical for decidualization in mice. This study has demonstrated for the first time that IL-11 regulates changes in the uterine extracellular matrix that are necessary for decidualization. The application of cDNA microarray analysis has revealed that lack of IL-11 signalling in *IL11Ra*^-/- ^mice results in differences in mRNA expression compared to wild type during artificial decidualization. Each of the ECM molecules investigated further in this study, collagen III, biglycan, nidogen-1 and SPARC, showed protein expression patterns consistent with a role in decidualization, with immunostaining in endometrial stromal cells and their surrounding matrices. Collagen III and biglycan were more abundant during defective decidualization in *IL11Ra*^-/- ^uterus, both at the mRNA and protein levels. This indicates that the cellular processes of decidualization including proliferation, differentiation, signal transduction and apoptosis may be facilitated by decreased expression of these matrix molecules.

As well as providing a dynamic structural framework, the ECM of the endometrium interacts with its associated cells to mediate critical processes, including adhesion, migration and differentiation (reviewed in [35]). Growth factor availability can also be regulated by binding to ECM components [36]. Collagens, elastin, structural glycoproteins (eg. fibronectin, laminin, SPARC and nidogen), proteoglycans (eg. biglycan) and glycosaminoglycans are the major components of endometrial matrix, and can act as ligands for both cell-cell and cell-matrix interactions [37]. Decidualization of endometrial stromal cells is associated with dramatic changes in matrix composition, including the phagocytosis and digestion of collagen fibrils, an increase in collagen fibril diameter [38], deposition of basement membrane proteins [39], the synthesis and secretion of sulfated glycosaminoglycans into the extracellular space and a decrease in elastic fibrils surrounding mature decidual cells [1]. Disruptions to the composition of uterine ECM during decidualization may be responsible for the failure of implantation in *IL11Ra*^-/- ^mice.

While the microarray data showed consistent, reproducible upregulation of COL3A1, BGN, SPARC and NID1 in *IL11Ra*^-/- ^compared to wild type uterus, this effect was not statistically significant when real-time RT-PCR was used as an alternative quantitation method. Many factors may contribute to discrepancies between cDNA microarray and real-time RT-PCR data. There are major differences in the approach to mRNA quantitation used by the two techniques. Using cDNA microarray, the mean fluorescence intensity ratio for each gene in *IL11Ra*^-/- ^or *IL11Ra*^+/+ ^uterus was calculated relative to a reference pool, and the ratio of *IL11Ra*^-/- ^to *IL11Ra*^+/+ ^determined by the use of computational algorithms. When quantitating the same mRNA species by real-time RT-PCR, a standard curve of known concentration was used to infer the absolute abundances of mRNA in the *IL11Ra*^-/- ^and *IL11Ra*^+/+ ^samples, which were then normalized for RNA input.

Real-time RT-PCR was chosen for cDNA microarray validation in this study because it has higher sensitivity and lower RNA requirements than Northern blot, but the lack of agreement between the two methods is not unusual. It is well recognized that fold change values for a given gene may vary widely, even between two different microarray techniques [40-43]. In using real-time RT-PCR to evaluate microarray data, Rajeevan et al [44] found that the majority of the array data were qualitatively accurate, but it was not possible to consistently validate genes showing less than a 4-fold difference on the array. Each of the genes examined in this study showed less than a 3-fold difference. It is not known how well array data correlates overall with data from RT-PCR or any other mRNA quantitation method [45], further complicating the interpretation of conflicting results.

There are a number of compelling arguments both for and against conducting corroborative studies for microarray data, and there is good evidence that the data is highly reliable when the experimental design and statistical analysis is sound [46]. In assessing the validity of the microarray data in this study, it is important to note that immunostaining for both collagen III and biglycan protein confirmed the differential expression seen by microarray analysis. This is striking, given that changes in protein expression detected by tissue microarray have been found to correlate with the mRNA change less than 50% of the time [45]. Given the cellular heterogeneity of the uterus, the localization of cell-specific expression is essential in extending microarray data on whole uterus to the investigation of decidualization. Neither SPARC nor nidogen-1 proteins were altered in expression by the absence of IL-11 signaling, but there may well be a delay between the mRNA and corresponding protein changes. This would not have been detected by using samples collected at the same time point for both mRNA and protein analyses.

For each of the genes examined, upregulation during defective decidualization in *IL11Ra*^-/- ^uterus is supported by existing data in the literature. Collagen III is a fibrillar collagen with known roles in differentiation and migration [47], and together with collagen I forms the structural support for the endometrium during the establishment of pregnancy [48]. Consistent with a role in decidualization, collagen III is both secreted and phagocytosed by mouse decidual cells [49], and secreted by first trimester decidual cells in the human [50]. The reduced number of collagen fibrils surrounding endometrial stromal cells in early pregnancy [51] may facilitate decidual transformation and blood vessel development [52]. This decrease has previously been detected primarily in the subepithelial endometrial stroma at day 4 [53]. In the rat, low levels of collagen III have been reported in the primary decidual zone, with much higher concentrations in the outer stroma and myometrium as decidualization progresses [54]. This is consistent with immunohistochemical data obtained for wild type mice in this study, showing very low intensity staining in the antimesometrial decidua, and higher intensity in the outer compartments of the uterus.

During the human menstrual cycle, collagen III immunostaining is higher in the proliferative compared to the secretory phase [55], indicating that downregulation and/or metabolism and redistribution of collagen III occurs with the onset of endometrial receptivity. Compared to proliferative phase endometrium, the ratio of collagen III to collagen I is decreased in decidual cells [56]. Aplin et al [55] observed changes in collagen III distribution from dense fibrils in the proliferative phase to matrix channels between decidual cells in the secretory phase. This may be involved in maintaining tissue integrity as the level of hydration increases, and in supporting movement of leukocytes through the tissue [57]. Defects in any of these processes in *IL11Ra*^-/- ^mice could contribute to impaired decidualization.

Using microarray analysis, the mRNA encoding procollagen III α1 (COL3A1) has been previously shown to decrease in abundance in the mouse uterus at estrus [58], and between days 3.5 and 5.0 of gestation [59], and to increase following ovariectomy in the rat [60]. Together with data from this study showing increased COL3A1 mRNA and mature collagen III protein in *IL11Ra*^-/- ^uterus at 48 h of decidualization, it appears that successful decidualization involves downregulation of COL3A1 transcription.

Biglycan is a small leucine-rich proteoglycan, which binds to type I [61] and type V collagen fibrils, transforming growth factor-β [62] and tumour necrosis factor-α [63] in vitro. Its function in ECM has not been well defined, but biglycan is thought to be involved in the control of cell migration [64]. In the wild type mouse uterus, there is low endometrial biglycan expression post-implantation [65]. Biglycan mRNA expression has been shown by oligonucleotide microarray to be downregulated in the secretory compared to the proliferative phase of the menstrual cycle in human endometrium [66,67], coincident with the window of implantation. As defective decidualization in *IL11Ra*^-/- ^mouse uterus was associated with the upregulation of biglycan mRNA, the activity of this proteoglycan in the ECM may inhibit the decidual response.

Decidual cells are known to express nidogen-1 as part of the pericellular basement-membrane laid down during decidualization [39]. The main function of nidogen in the basement-membrane is to connect networks of collagen IV and laminin [68], but nidogen also binds perlecan [69], fibulins [70] and fibronectin [71]. Changes in nidogen mRNA levels have been reported during the establishment of the placenta in the mouse, with in situ hybridization revealing highly restricted expression in decidual and maternal endothelial cells [72]. This study has now shown much earlier nidogen-1 protein expression in the decidual cells, glandular epithelial cells and epithelial basement membrane of the artificially induced deciduoma, and indicated aberrant increased expression of the NID1 gene during defective decidualization.

SPARC (osteonectin/BM-40) is described as a matricellular glycoprotein, in that it binds to both cells and ECM to regulate cell-matrix interactions [73]. Like other matricellular proteins, SPARC can bind and alter the activity of cytokines and induce the expression of proteinases and their inhibitors [74]. SPARC is often expressed in tissues undergoing cell proliferation, migration and ECM remodeling [75], so it is not surprising that substantial expression of SPARC has been observed in human decidua [76]. Differences in immunostaining intensity have been associated with the degree of decidualization, with the strongest staining seen in the cytoplasm of decidualizing cells, decreasing as decidualization progresses [76]. Fully decidualized cells were found to express SPARC pericellularly, indicating a role in mediating interactions of decidual cells with their surrounding matrix. Binding of SPARC to a number of ECM components, including collagen III and nidogen, may contribute to the structural integrity of the tissue [77]. It could therefore be hypothesized that during normal decidualization, SPARC, collagen III and nidogen-1 are coordinately downregulated to allow loosening of the tissue in preparation for trophoblast invasion. In both models of IL-11Rα deficiency [7,8], implantation sites have increased rather than decreased numbers of invading trophoblast giant cells. This pathological invasion is thought to occur subsequent to failure of decidualization, highlighting the importance of tight regulation of ECM components in normal decidual function.

Using mRNA and protein expression studies alone, it is not possible to determine whether IL-11 affects ECM molecule expression directly or indirectly. The presence of STAT binding sites in the promoters for COL3A1, BGN, NID1 and SPARC would confirm that a direct interaction is possible, but would not establish that the interaction is occurring in the uterus during decidualization. Given that IL-11 is a secreted cytokine with autocrine and paracrine activity, a direct effect on ECM molecule transcription would be dependent on coincident expression patterns of the ECM molecules, IL-11 and its receptors. Maximal expression of IL-11 and IL-11Rα mRNA has been reported in the predecidual and decidual cells of the mouse implantation site by in situ hybridization [7]. Expression of gp130 mRNA is detectable in the glandular epithelium from days 3 – 7, and in decidual cells from day 5 [78]. Given that the ECM molecules investigated in this study are more widely expressed in the uterus, it is likely that regulation by IL-11 is indirect.

Indirect effects of IL-11 on ECM composition could be mediated by matrix metalloproteinases (MMPs) and/or their inhibitors. Despite their presence on the NIA 15K microarray, differential expression was not observed for MMP-2, MMP-9, TIMP-2, or TIMP-3 in the *IL11Ra*^-/- ^uterus compared to wild type. Previous in vitro studies have indicated that IL-11 inhibits MMP-1 and -3 protein in human synovium [79], and enhances the ability of mouse osteoblasts to synthesize MMPs responsible for the degradation of collagen I [80]. IL-11 does not influence the activity of stromelysin in human chondrocytes [13], or induce MMP-2, -7 or -9 in human endometrial epithelial or stromal cells [81], but tissue inhibitor of metalloproteinases (TIMP)-1 is known to be induced by IL-11 in vitro [13]. While not directly supporting a role in ECM degradation, these interactions suggest that IL-11 is involved in regulating the balance between MMP and TIMP activity in the tissue.

## Conclusions

This investigation of the downstream targets of IL-11 during mouse decidualization has uncovered previously unknown interactions between IL-11 and uterine ECM composition. Dysregulation of collagen III, biglycan, nidogen-1 and SPARC in the absence of IL-11 signaling at the time of decidualization may indicate essential functions for these molecules during the implantation process in mice. Functional studies using mouse and human endometrium may further clarify the mechanisms of IL-11 action on the ECM during this critical time in embryo implantation. By elucidating the role of IL-11 regulated genes in decidualization, future work may identify potential new targets for the manipulation of human fertility.

## Authors' contributions

CAW carried out all the experimental work and statistical analysis and drafted the manuscript. LR provided the heterozygote *IL11Ra*^+/- ^breeding pairs. LAS conceived of the study, participated in its design and coordination and helped to draft the manuscript. All authors read and approved the final manuscript.
